# Berry Population Analysis: Atomic Charges from the Berry Curvature in a Magnetic Field

**DOI:** 10.1021/acs.jctc.2c01138

**Published:** 2023-01-27

**Authors:** Laurens D. M. Peters, Tanner Culpitt, Erik I. Tellgren, Trygve Helgaker

**Affiliations:** Hylleraas Centre for Quantum Molecular Sciences, Department of Chemistry, University of Oslo, P. O. Box 1033, Blindern, N-0315Oslo, Norway

## Abstract

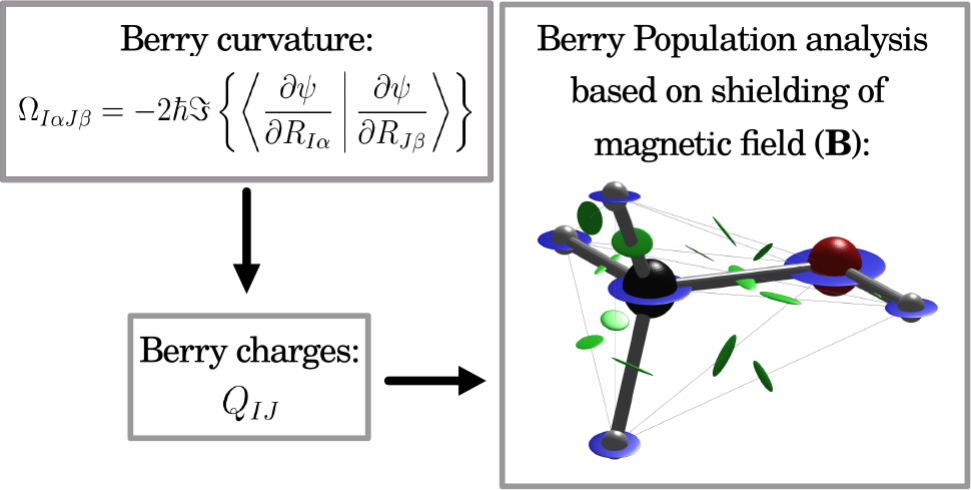

The Berry curvature is essential in Born–Oppenheimer molecular dynamics, describing the screening of the nuclei by the electrons in a magnetic field. Parts of the Berry curvature can be understood as the external magnetic field multiplied by an effective charge so that the resulting Berry force behaves like a Lorentz force during the simulations. Here, we investigate whether these effective charges can provide insight into the electronic structure of a given molecule or, in other words, whether we can perform a population analysis based on the Berry curvature. To develop our approach, we first rewrite the Berry curvature in terms of charges that partially capture the effective charges and their dependencies on the nuclear velocities. With these Berry charges and charge fluctuations, we then construct our population analysis yielding atomic charges and overlap populations. Calculations at the Hartree–Fock level reveal that the atomic charges are similar to those obtained from atomic polar tensors. However, since we additionally obtain an estimate for the fluctuations of the charges and a partitioning of the atomic charges into contributions from all atoms, we conclude that the Berry population analysis is a useful alternative tool to analyze the electronic structures of molecules.

## 1 Introduction

Population analysis is one of the simplest and most common tools of a quantum chemist to gain insight into the electronic structure of a molecular system.^1−4^ The central idea is that we can assign a partial or atomic charge *q*_*I*_ to every atom *I*, allowing us to analyze the bonding situation or to make predictions regarding the reactivity of a compound, avoiding a more complex analysis of the electronic density.

Today, a large number of population analyses exist, each with its own pros and cons. Many methods rely on a partitioning of either the wave function or the electron density into atomic fragments. Prominent examples are the Mulliken,^5−7^ Löwdin,^8,9^ natural,^10^ Bader,^11^ and Hirshfeld^12^ charges. While the first two use atom-centered basis functions to determine an atomic contribution to the wave function, the remaining directly determine atomic densities that add up to the total electronic density. At this point, we should mention the Charge Model 5 (CM5^13^) and the Density Derived Electrostatic and Chemical (DDEC6^14,15^) charges as significant improvements on these methods.

The second large group of population analyses tries to access the atomic charges via observables of the molecule. Restrained Electrostatic Potential (RESP) charges^16^ are, for example, extracted from the electrostatic potential of the molecule, whereas the atomic-polar-tensor or Born effective charges are determined as derivatives of the electronic dipole moment with respect to nuclear displacements.^17−21^

Here, we introduce a new population analysis that fits into the second group discussed above. The idea behind our method is simple: Any charged particle moving in a magnetic field **B** with velocity **Ṙ**_*I*_ experiences a Lorentz force **F**_*I*_^L^ inducing a cyclic motion about the magnetic field vector,
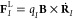
1Consequently, when the forces acting on the nuclei of a molecule in a magnetic field are known, we can use these to determine atomic charges.

The *ab initio* calculation of forces of molecules in a magnetic field is, however, not straightforward; only very recently^22,23^ were the first simulations conducted. They require a nonperturbative treatment of the magnetic field^24−40^ and the use of London orbitals^41−44^ to ensure the correct physics—namely, that all observables are gauge and translationally invariant^45^ and that neutral atoms and molecules do not “feel” an overall Lorentz force in a magnetic field.^46^ In a magnetic field, each nucleus experiences not only the usual Lorentz force, but also the Berry force, generated by the electrons in the system^47−53^
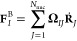
2where **Ω**_*IJ*_ is the Berry curvature,^54−57^ representing the screening of the nuclei by the electrons. It contains derivatives of the electronic wave function with respect to the nuclear coordinates and can be determined at the Hartree–Fock (HF) level of theory, via a numerical scheme^45^ or by solving the coupled-perturbed HF equations.^58^

In this work, we demonstrate how **Ω**_*IJ*_ can be used as a population analysis of a given molecule. After a short discussion of its calculation and properties (Section 2.1), we rewrite the Berry curvature in terms of polarization tensors (Section 2.2) and Berry charges (Section 2.3). The latter give a simple picture of the screening process captured by the Berry curvature (Section 2.4), which we use as a justification for the Berry population analysis (Section 2.5). Having summarized the computational details in Section 3, we analyze the Berry charges and validate the resulting population analysis via comparison to the established Mulliken^5^ and atomic-polar-tensor^17^ charges in Section 4. Conclusions and future directives are given in Section 5.

## 2 Theory

We use indices *a*, *b*, ... for Cartesian components, indices *I*, *J*, ... for the *N*_nuc_ nuclei, and indices *i*, *j*, ... for the *N*_occ_ occupied molecular orbitals {φ_*i*_(**r**; **R**, **O**, **B**)}. The electronic and nuclear coordinates are denoted by **r** and **R**, respectively, while **p̂** and **P̂** refer to the corresponding canonical momentum operators:
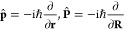
3We use *eZ*_*I*_, *M*_*I*_, **R**_*I*_, and **Ṙ**_*I*_ to represent the charge, mass, coordinates, and velocity of nucleus *I*. Here, **O** is the gauge origin and **B** a uniform magnetic field. By introducing the magnetic field tensor
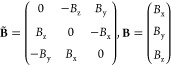
4we may reformulate a cross product of **B** with a vector **v** as a matrix multiplication **B** × **v** = **B̃v**. We also define the Jacobian matrix for derivatives of vectors with respect to a nuclear coordinate by
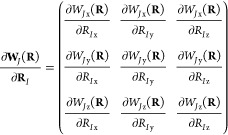
5For brevity, we drop the dependence of operators, expectation values, and orbitals on **O** and **B**.

### 2.1 Berry Curvature in a Magnetic Field

The force on atom *I* in a magnetic field consists of the Born–Oppenheimer force, the Lorentz force, and the Berry force:^22,45,49,53,58^
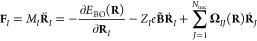
6Here, *E*_BO_(**R**) is the potential energy with or without the diagonal Born–Oppenheimer correction (DBOC) included, and **Ω**_*IJ*_(**R**) is the Berry curvature, which is determined from derivatives of the geometric vector potential [**χ**_*I*_(**R**)] with respect to the nuclear coordinates:
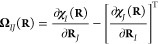
7In HF and density-functional-theory (DFT) calculations, both quantities are calculated from derivatives of the occupied molecular orbitals with respect to the nuclear coordinates:
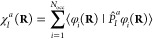
8
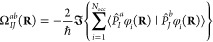
9

More details on their interpretations and calculations from London orbitals are given elsewhere.^22,45,46,49,53,58^ Here, we note that the geometric vector potential is real valued and gauge dependent, while the Berry curvature has the units [*B*_0_*e*] (magnetic field strength times electronic charge) and is linked to the screening of the nuclear charges by the electrons in a magnetic field. The components of the Berry curvature obey the magnetic-translational sum rule^46,49^
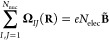
10and are, by construction, antisymmetric upon permutation of nuclei:
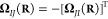
11Using the latter property, we can separate each component into parts that are permutationally symmetric (*+*) and antisymmetric (−), respectively:
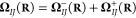
12
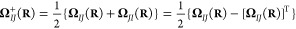
13
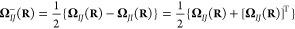
14

The unit of the Berry curvature and of eq 10 indicate that (at least parts of) **Ω**_*IJ*_(**R**) can be interpreted as the external magnetic field multiplied with an effective atomic charge,
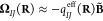
15where all charges sum up to the total number of electrons:
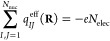
16Our task is therefore now to rewrite or approximate the Berry curvature in terms of these charges. This is done in two steps: (1) by separating **Ω**_*IJ*_(**R**) into a magnetic-field-dependent part and a polarization tensor (Section 2.2), and (2) by reducing the latter to charges (Section 2.3). Having analyzed and visualized these charges (Section 2.4), we construct our population analysis (Section 2.5).

### 2.2 Polarization-Tensor Approximation of the Berry Curvature

Let us assume that the geometric vector potential can be approximated in the following manner:
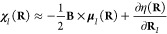
17Here, η(**R**) is an arbitrary gauge function, and we refer to **μ**_*I*_(**R**) as a *nuclear contribution* to the total electronic dipole moment μ(**R**):
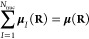
18This relation ensures the correct translational behavior of the total geometric vector potential [∑_*I* = 1_^*N*_nuc_^**χ**_*I*_(**R**), see ref (46)] and coincides with the dipolar sum rule introduced by Zabalo, Dreyer, and Stengel in ref (59). Note the analogy between our formulation of **χ**_*I*_(**R**) and the external vector potential in the Coulomb gauge [**A**_*I*_(**R**)] as well as the vector potential of a single London orbital centered at **R**_*I*_ [**χ**_LDN_(**R**_*I*_)]:^45^
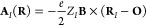
19
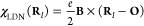
20

Inserting our ansatz into eq 7 and introducing the polarization tensor,
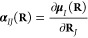
21we obtain a compact expression that separates the *explicit* magnetic field dependence (**B**, [*B*_0_]) from the electronic structure dependence (**α**_*IJ*_(**R**), [*e*]) that depends only *implicitly* on **B**:
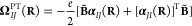
22From now on, we refer to **Ω**_*IJ*_^PT^(**R**) as the polarization-tensor approximation of the Berry curvature **Ω**_*IJ*_(**R**) ≈ **Ω**_*IJ*_^PT^(**R**). Note that contributions from the gauge functions vanish, since
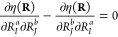
23

The approximate Berry curvature **Ω**_*IJ*_^PT^(**R**) retains all the important properties of the exact Berry curvature: (1) the permutational antisymmetry [see eq 11], (2) the near-linear dependence on the magnetic field strength, and (3) the magnetic-translational sum rule [see eq 10]. The latter property can be easily demonstrated by writing out the sum over all polarization tensors:
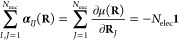
24This underscores the relation between the dipolar^59^ and the magnetic-translational^46^ sum rule. It also shows that **α**_*IJ*_(**R**) can be regarded as a *nuclear contribution* to the atomic polar tensor **V**_*J*_(**R**):
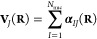
25Note that the polarization-tensor approximation of the Berry curvature in eq 22 has been used previously in the M3 model in ref (46), where **μ**_*I*_(**R**) was obtained from the Mulliken partitioning scheme. Here, we do not assume a *specific* form for **μ**_*I*_(**R**) or **α**_*IJ*_(**R**), but use their properties and physical interpretations to gain insight into the Berry curvature itself.

As a final step of the subsection, we write out the permutationally symmetric (*+*) and antisymmetric parts (−) of **Ω**_*IJ*_^PT^(**R**),
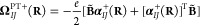
26
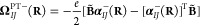
27in terms of the corresponding components of the polarization tensor:
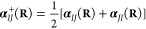
28
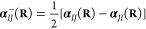
29

### 2.3 Charge Approximations to the Berry Curvature

The polarization-tensor approximation to the Berry curvature can be simplified further when we reduce the polarization tensors to a few meaningful components or charges. Here, we focus on two charges: *Q*_*IJ*_(**R**) and *P*_*IJ*_(**R**).

We start by only taking into account the *isotropic* part [*Q*_*IJ*_(**R**)] of the permutationally symmetric polarization tensor:
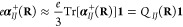
30This approximation is exact for atoms and dissociated molecules, where the electrons screen the nuclear charges isotropically:
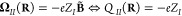
31It may hold also for highly symmetric molecular systems. From eqs 24 and 28, we know that these charges are permutationally symmetric and sum up to the total number of electrons:
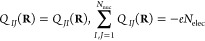
32

Additionally, we may reduce the permutationally antisymmetric polarization tensor to its component *P*_*IJ*_(**R**) along the normalized interatomic distance vector **R̅**_*IJ*_:
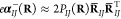
33where
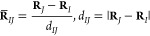
34This approximation is exact when the contributions to the electronic dipole moment **μ**_*I*_(**R**) and **μ**_*J*_(**R**) depend solely on *d*_*IJ*_, while their sum is aligned with **R̅**_*IJ*_:
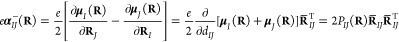
35We therefore expect it to hold for linear molecules with cylindrical symmetry and a nonzero electric dipole moment. Since **α**_*IJ*_^–^(**R**) is permutationally antisymmetric and adds up to zero when summed over all pairs of nuclei, the same relations hold for *P*_*IJ*_(**R**):
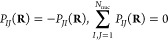
36

Equipped with eqs 30 and 33, we now construct two charge approximations, termed B1 and B2, to the Berry curvature:
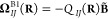
37
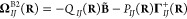
38where **Γ**_*IJ*_^+^(**R**) depends only on the orientation of the molecule relative to the magnetic field:
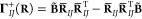
39Analyzing the permutational symmetry of the B2 model, we see that *Q*_*IJ*_(**R**) and *P*_*IJ*_(**R**) recover the symmetric and antisymmetric parts, respectively:
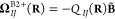
40
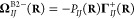
41We can therefore also set up the charge approximations by invoking the decomposition of a matrix into a scalar (*s*_*IJ*_), a vector (**V**_*IJ*_), and a traceless matrix (**T**_*IJ*_):^60^
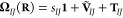
42In this case, −*Q*_*IJ*_(**R**) can be interpreted as the component of **V**_*IJ*_ along **B**, whereas *P*_*IJ*_(**R**)**Γ**_*IJ*_^+^(**R**) is the component of **T**_*IJ*_ constructed from the orthogonal axes **R̅**_*IJ*_ and **B** × **R̅**_*IJ*_.

Note that the B2 model is exact for diatomic molecules oriented perpendicular to the magnetic field.^45^ With the magnetic field aligned with the *z*-axis, the Berry curvature then takes the following simple form, which is perfectly captured by the B2 approximation and its two charges:
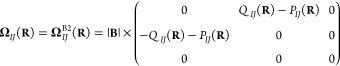
43The *Q*_*IJ*_(**R**) charges are thus identical to the screening charges introduced in refs (45) and (61). The contribution from the *P*_*IJ*_(**R**) charges in eq 42 vanishes for H_2_ or when we assume that the diatomic system is aligned with the magnetic field. For these systems, the B1 model correctly reproduces the behavior of the exact Berry curvature:
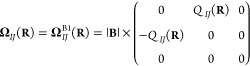
44

Even for a general molecule, the B1 and the B2 models may be useful alternatives to the full Berry curvature, especially when conducting molecular dynamics simulations in magnetic fields. The remaining challenge is, however, to determine the *Q*_*IJ*_(**R**) and *P*_*IJ*_(**R**) charges without calculating the Berry curvature. Such an attempt was made in the M2 model of ref (46), where the *Q*_*IJ*_(**R**) charges were replaced by Mulliken overlap populations. Here, we focus on the physical interpretation of the Berry curvature via the charges *Q*_*IJ*_(**R**) and *P*_*IJ*_(**R**).

### 2.4 Interpretation of Berry Charges and Charge Fluctuations

The most straightforward way to understand the role of *Q*_*IJ*_(**R**) and *P*_*IJ*_(**R**) follows from the special case of diatomic molecules perpendicular to the field. When the molecule and the magnetic field are aligned with the *x*- and *z*-axes, respectively, the Berry curvature takes the form of eq 42, and we can write the Cartesian components of the Berry force as
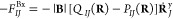
45
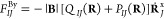
46
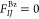
47where **F**_*IJ*_^B^ is the Berry force on atom *I* induced by the movement of atom *J*. Equations 45 and 46 can be interpreted as Lorentz forces with *Q*_*IJ*_(**R**) and *P*_*IJ*_(**R**) serving as charges. However, the signs of these charges depend on the direction of the velocity.

To investigate this further, it is helpful to expand the squared norm of the Berry force as
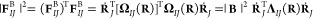
48where **Λ**_*IJ*_(**R**) = |**B**|^–2^ [**Ω**_*IJ*_(**R**)]^T^**Ω**_*IJ*_(**R**) is a symmetric, positive semidefinite matrix. It can therefore be diagonalized,
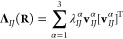
49and the eigenvectors **v**_*IJ*_^α^ may be thought of as the directions of the semiaxes of an ellipsoid. The eigenvalues satisfy λ_*IJ*_^α^ ≥ 0, and  are the lengths of the semiaxes. Hence, we may visualize the effect of **Ω**_*IJ*_(**R**) on a velocity **Ṙ**_*J*_ by plotting the ellipsoid:
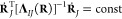
50Since one eigenvalue of **Λ**_*IJ*_(**R**) is zero, the ellipsoid is a flat disk, and we interpret [**Λ**_*IJ*_(**R**)]^−1^ as a generalized inverse. In the context of the Berry force and in line with our starting point in eq 15, λ_*IJ*_^α^ can be interpreted as the square of an *effective charge q*_*IJ*_^eff,α^(**R**) that weights the Lorentz force due to a velocity **Ṙ**_*J*_ along **v**_*IJ*_^α^.

**Figure 1:**
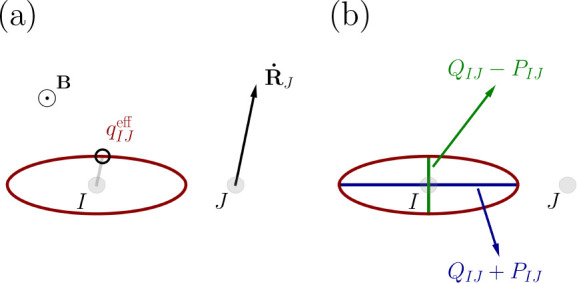
Geometric illustration of the directional dependence of the effective charge on *I* (*q*_*IJ*_^eff^) from the velocity of *J* (**Ṙ**_*J*_) for a diatomic molecule perpendicular to the magnetic field **B**. (a) The radius of the ellipse in the direction of **Ṙ**_*J*_ corresponds to *q*_*IJ*_^eff^. (b) The shape of the ellipse is determined by the Berry charges and charge fluctuations.

In the special case of a linear molecule perpendicular to the field, **Λ**_*IJ*_(**R**) is a diagonal matrix from which we can directly determine the eigenvalues as
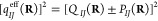
51The eigenvalue along the *z*-axis is zero, so that our ellipsoid of eq 50 collapses to an ellipse with *Q*_*IJ*_(**R**) ± *P*_*IJ*_(**R**) as principle axes along the *x*- and *y*-directions, respectively (see Figure 1). From this simple picture, we see that *Q*_*IJ*_(**R**) is the *isotropic* component of the charge, being independent of the direction of the velocity. This observation agrees with our conclusion in ref (45)—namely, that *Q*_*IJ*_(**R**) represents the amount of electrons by which nucleus *J* screens nucleus *I* or *vice versa*. For this reason, we refer to the *Q*_*IJ*_(**R**) as the Berry charges from now on. The *P*_*IJ*_(**R**) can be understood as a measure of *anisotropy*—that is, the amount by which the effective charge fluctuates with direction of the velocity. We therefore refer to the *P*_*IJ*_(**R**) as Berry charge fluctuations.

Let us now consider a molecule with an arbitrary angle θ between **R̅**_*IJ*_ and **B**. In the Appendix, we show that, using the B2 model for the Berry curvature, we obtain the following two nonzero eigenvalues:
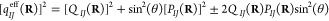
52This demonstrates that the contribution of the Berry charge fluctuations depends on θ. For a molecule parallel to the magnetic field, the effective charge becomes isotropic, while an angle of π/2 reproduces the result in eq 51:
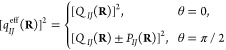
53In the latter case, the eigenvectors are aligned with **R̅**_*IJ*_ and **B** × **R̅**_*IJ*_, respectively. It should be noted that the exact **Λ**_*IJ*_(**R**) may have different semiaxes than assumed in the B2 approximation.

### 2.5 Berry Population Analysis

As a final step in this section, we introduce a new population analysis, which we refer to as Berry population analysis. In general, such an analysis aims at extracting atomic charges from quantum-chemical calculations, yielding insights into bonding situations, reactivity, and so on. Prominent examples are the Mulliken charges^5^ [*q*_*I*_^M^(**R**)] and overlap populations [*Q*_*IJ*_^M^(**R**)], which use molecular orbitals constructed only from basis functions assigned to atom *I* [φ_*i*_^(*I*)^(**R**)]:
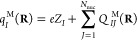
54
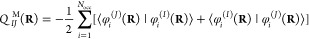
55and the generalized atomic-polar-tensor charges^17^ [*q*_*I*_^D^(**R**)], which determine the isotropic part of the atomic polar tensor:
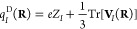
56

In the previous subsections, we have shown that the Berry curvature can indeed be interpreted as an effective charge. However, since the effective charge depends on the direction of the velocity, there is no unique definition of an atomic charge. Similar to other works,^45,61^ we define the *Berry atomic charge* [*q*_*I*_^B^(**R**)] as the sum over all isotropic Berry charges and the nuclear charge:
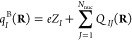
57This choice ensures that the charge is isotropic and corresponds to the effective charge on *I* during a rigid translation of the entire molecule **Ṙ**_T_:
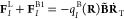
58

We note that the Berry population analysis requires the presence of a magnetic field. This should be kept in mind when comparing to population analyses performed at zero field. Additionally, it should be mentioned that the electron density depends on the orientation of the molecule with respect to the magnetic field vector. To account for this indirect dependence, every population analysis in a nonzero field requires rotational averaging ⟨*q*_*I*_^B^(**R**)⟩_rot_ before evaluation.

## 3 Computational Details

All calculations presented here were performed at the Hartree–Fock/l-cc-pVDZ level of theory using the London(62) program package, at the zero-field optimized molecular geometries. Here l-cc-pVDZ denotes the London-orbital variant of the contracted cc-pVDZ^63^ basis set, which has been shown to give Berry curvatures in good agreement with results obtained from the computationally more expensive l-cc-pVTZ basis set.^46^ The magnetic field strength was set to 0.001*B*_0_. The Berry curvature and the atomic-polar-tensor charges were obtained from finite differences calculations with a step size of 5 × 10^–4^ Bohr. The error of this numerical approach is less than 0.01%.^46^ The Berry charges and charge fluctuations were subsequently obtained by solving eqs 40 and 41 in a least-squares fashion:
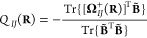
59
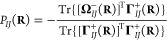
60

From these quantities, we calculate the Berry atomic charges *q*_*I*_^B^(**R**) and the corresponding approximate Berry curvatures **Ω**_*IJ*_^B1^(**R**) and **Ω**_*IJ*_^B2^(**R**). The Mulliken charges *q*_*I*_^M^(**R**), overlap populations *Q*_*IJ*_^M^(**R**), and Mulliken approximation to the Berry curvature **Ω**_*IJ*_^M2^(**R**) (see ref (46)) were obtained from single-point calculations. As in ref (46), we quantify the error of an approximate Berry curvature by the following “screening error per electron”:
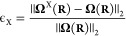
61For the B2 approximation, we also determine ϵ_S_^B2^, ϵ_V_^B2^, and ϵ_T_^B2^ as the errors within the scalar, vector, and tensor parts of the Berry curvature (see eq 42):
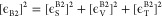
62

For the rotationally averaged values ⟨*q*_*I*_^X^(**R**)⟩_rot_, ⟨*Q*_*IJ*_(**R**)⟩_rot_, and ⟨ϵ_X_⟩_rot_, we performed a numerical spherical integration over 146 geometries with different orientations relative to the magnetic field, using points and weights from the Python3.6 quadpy(64) package. As shown in Figure S1 in the Supporting Information, a grid of 146 points is sufficient to calculate the rotational average with an error below 0.1% for the current setup. The magnetic field is small enough (0.001*B*_0_) that we can use the same structure for all orientations. A more detailed discussion of the influence of magnetic field strength and basis set size on the Berry charges can be found in ref (61).

## 4 Results and Discussion

### 4.1 Validation of the Charge Approximations

**Figure 2:**
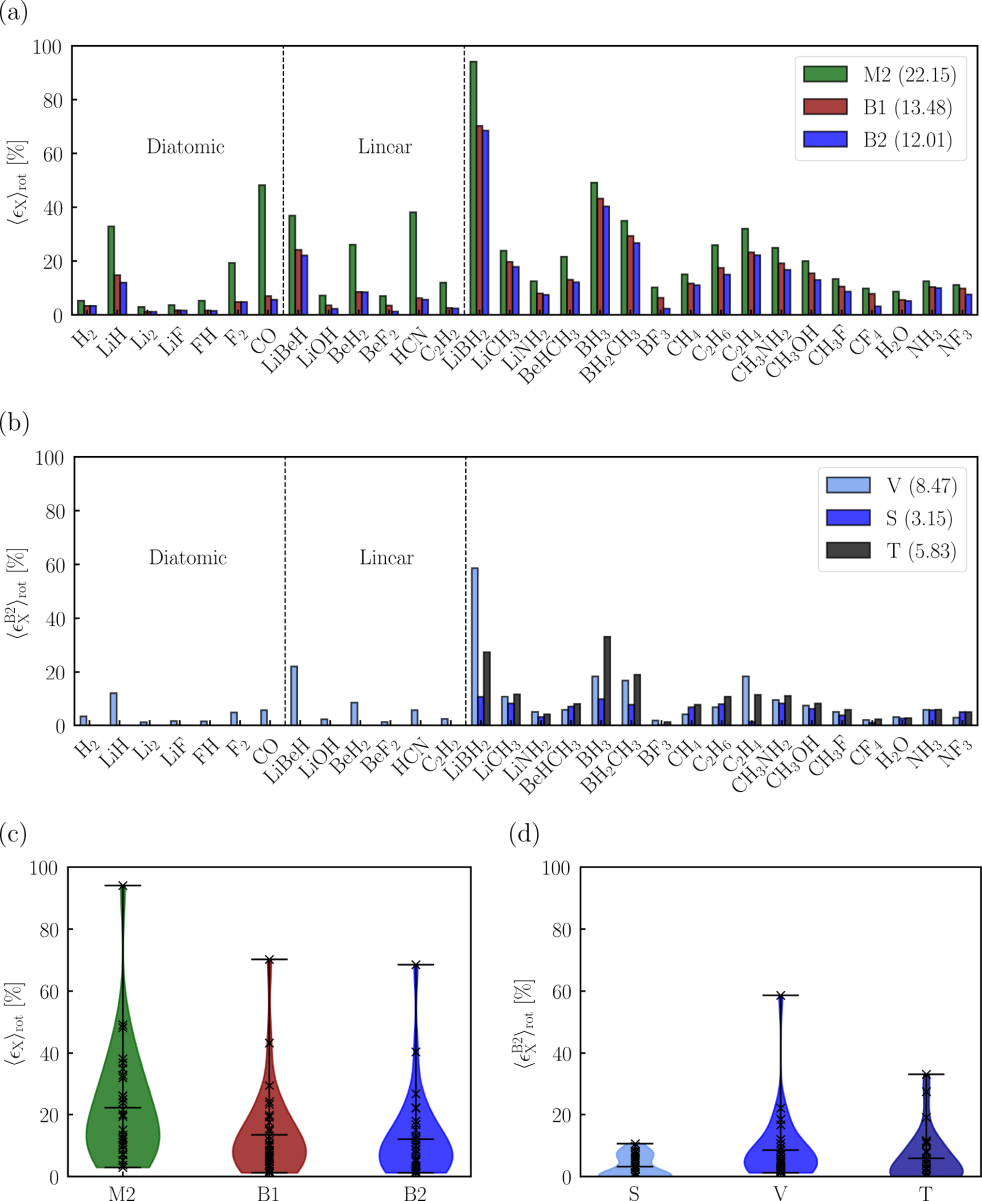
Rotationally averaged errors (see eq 61) of (a) the approximate Berry curvatures M2,^46^ B1 (eq 37), and B2 (eq 38) and (b) the scalar (S), vector (V), and tensor (T) components of the B2 approximation calculated for a series of molecules. The mean error of all molecules is given in brackets. In (c) and (d), we show violin plots for all the data points in (a) and (b), respectively.

Before discussing the Berry charges, charge fluctuations, and corresponding population analyses, we need to validate the charge approximations B1 (see eq 37) and B2 (see eq 38). The main question is how much of the exact Berry curvature is captured by *Q*_*IJ*_(**R**) and *P*_*IJ*_(**R**). As a measure of the screening error, we calculate the rotational average of ϵ_B1_ and ϵ_B2_ for a set of 30 molecules—see Figure 2, where we also show the error of the second Mulliken approximation (M2, see ref (46)) for comparison as well as a decomposition of ϵ_B2_ into contributions arising from the scalar, vector, and tensor component of the Berry curvature (see eq 62).

The new approximations perform significantly better than the Mulliken approximation [Figure 2(a)] For every molecule, B1 is closer to the exact Berry curvature, the average error decreasing from 22.15% for M2 to 13.48% for B1. This reduction is expected since the *Q*_*IJ*_(**R**) charges in the B1 model are chosen to minimize ϵ. Inclusion of the Berry charge fluctuations *P*_*IJ*_(**R**) reduces the error further to 12.01% for the B2 model. As expected from our derivation of the B2 model, this effect is especially strong for linear molecules, where the errors from the scalar and tensor contribution vanish. For nonlinear molecules, the error appears to be evenly spread among the different contributions [S, V, T in Figure 2(b)], indicating that it would require at least three additional parameters (or charges) to significantly reduce the error.

The violin plots in Figure 2(c, d) show that, even for the B2 approximation, there are several outliers—for example, LiH, BH_3_, LiBeH, and LiBH_2_. As discussed in ref (46), our charge models are probably less accurate for low-valent molecules. In general, we conclude that many features of the Berry curvature are well captured by the proposed Berry charges and charge fluctuations, which will therefore be a useful tool to study the effect of **Ω**(**R**) on molecular dynamics.

### 4.2 Berry Charges and Charge Fluctuations

**Figure 3:**
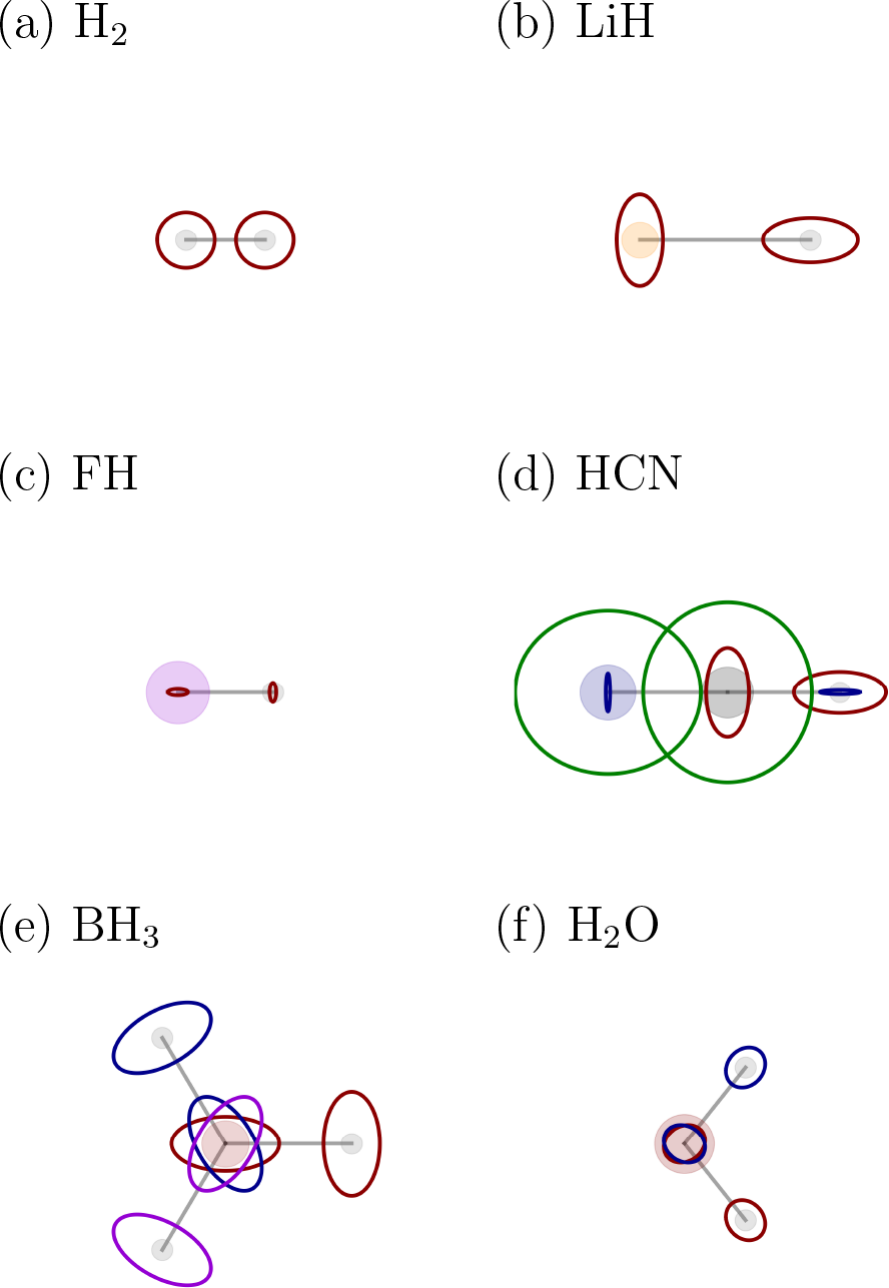
Visualization of the effective charges *q*_*IJ*_^eff^, obtained from the Berry charges and charge fluctuations, for a series of planar molecules perpendicular to the magnetic field. Ellipses representing different pairs *IJ* are shown in different colors, and the ellipse representing *IJ* is centered at **R**_*I*_. For brevity, we do not show the *q*_*II*_^eff^ in (a–f) and the *q*_HH_^eff^ in (e–f).

To investigate *Q*_*IJ*_(**R**) and *P*_*IJ*_(**R**), we consider a test set of eight small molecules (H_2_, LiH, BH_3_, CH_4_, HCN, NH_3_, H_2_O, and FH), representing a wide range of bond types, electronegativity differences, and orientations toward the magnetic field vector (see Tables 1 and 2). For the planar molecules in a perpendicular field orientation, the Berry charges are visualized in Figure 3 as discussed in Section 2.4.

**Table 1:** Berry Charges [*Q*_*IJ*_(**R**)] and Charge Fluctuations [*P*_*IJ*_(**R**)] of Linear Molecules H_2_, LiH, FH, and HCN in Parallel and Perpendicular Field orientations^a^

Mol.	**B**	*Q*_HH_(**R**)	*Q*_HX_(**R**)	*P*_HX_(**R**)
H_2_	⊥	–0.610	–0.390	0.000
	∥	–0.559	–0.441	0.000
LiH	⊥	–1.073	–0.478	–0.164
	∥	–1.674	–0.042	0.000
FH	⊥	–0.517	–0.090	0.046
	∥	–0.271	–0.290	0.000
HCN	⊥	–0.387	–0.307	–0.046
	∥	–0.570	–0.189	0.000

a For HCN, HX is the sum of the HC and HN contributions.

**Table 2:** Berry Charges [*Q*_*IJ*_(**R**)] and Charge Fluctuations [*P*_*IJ*_(**R**)] of Molecules of Type XH_*n*_ (X = B, C, N, O) with Different Orientations of the Principal Axis Relative to the Magnetic Field^a^

Mol.	**B**	*Q*_XX_(**R**)	*Q*_XH_(**R**)	*P*_XH_(**R**)
BH_3_	∥	–2.683	–1.667	–0.522
	⊥	–3.217	–1.294	–1.325
	⊥	–3.217	–1.294	–0.884
CH_4_	∥	–4.145	–1.800	–0.224
	⊥	–4.145	–1.800	–0.296
	⊥	–4.145	–1.800	–0.301
NH_3_	∥	–6.012	–1.212	0.057
	⊥	–6.474	–0.832	–0.156
	⊥	–6.474	–0.832	–0.293
H_2_O	∥	–8.168	–0.287	–0.393
	⊥	–7.948	–0.561	–0.206
	⊥	–7.825	–0.543	0.051

a XH denotes the sum over all hydrogens of the molecule.

We draw two main conclusions. First, the Berry charges and charge fluctuations depend strongly on the field orientation with CH_4_ being an exception due to its tetrahedral symmetry. Second, the Berry charges correlate with bonding properties of the corresponding molecule. The magnitude of *Q*_HX_(**R**) is larger when the HX bond is covalent (less than −0.4 for H_2_ and CH_4_) and smaller when it is considered to be more ionic (greater than −0.4 for H_2_O and NH_3_). Electronegativity also has an impact: as H becomes more electropositive, *Q*_HH_(**R**) increases as seen for LiH (−1.1/–1.7 in the perpendicular/parallel field orientation), H_2_ (−0.6/–0.6), and FH (−0.5/–0.3).

The Berry charge fluctuations are smaller than the Berry charges and may be positive and negative. Charge anisotropy occurs for every molecule except H_2_ in Figure 3—the largest anisotropies are observed for LiH, BH_3_, and HCN, where the shapes around the hydrogen atom have the largest deviation from a circle.

### 4.3 Berry Population Analysis

**Figure 4:**
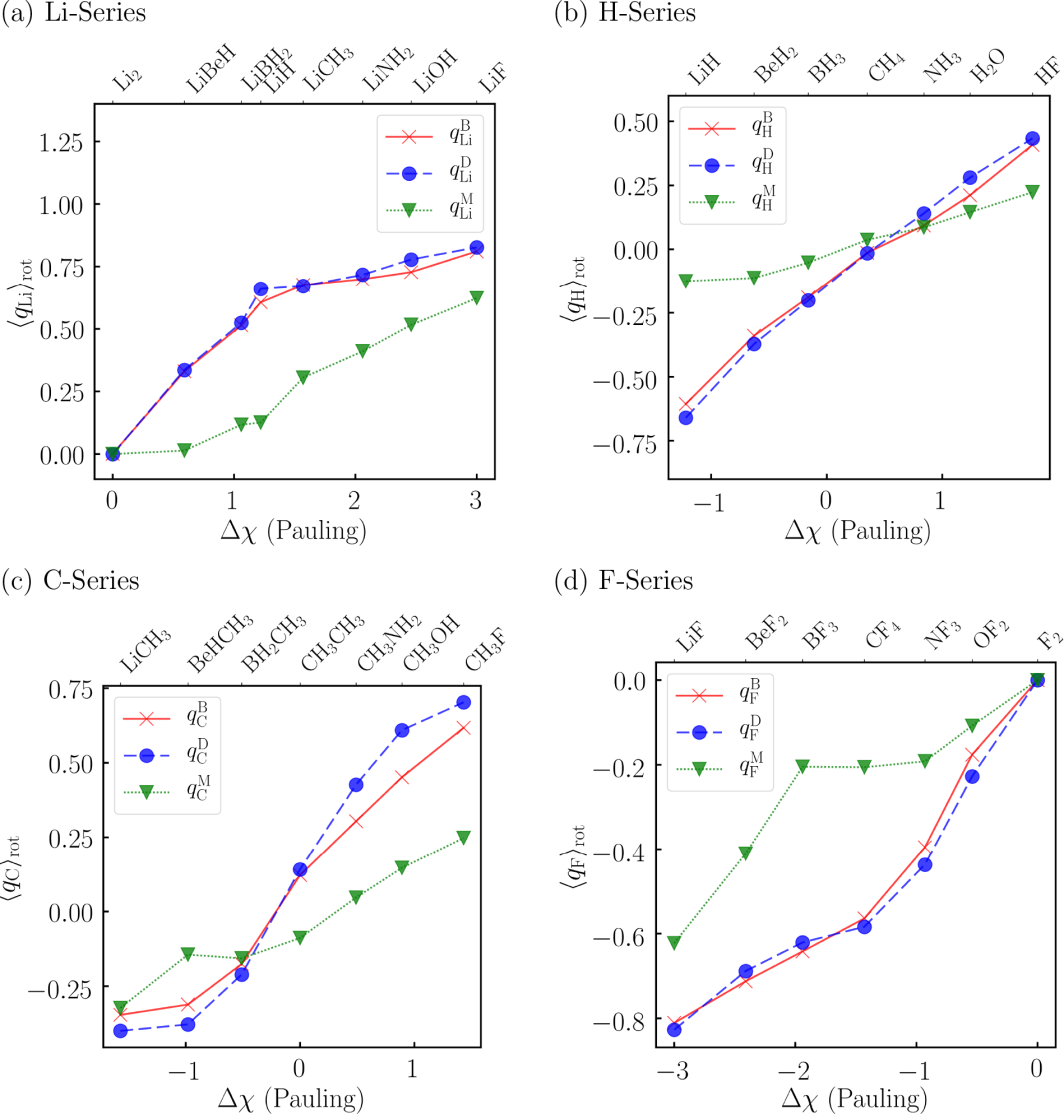
Comparison of rotationally averaged Berry (B), atomic-polar-tensor (D), and Mulliken (M) atomic charges (*q*_*I*_^B/D/M^, in *e*, see eqs 54, 56, and 57) for a series of Li-containing molecules (a), H-containing molecules (b), C-containing molecules (c), and F-containing (d) molecules. For each series, the molecules are sorted according to the Pauling electronegativity difference (*Δχ*) between the investigated atom (Li, H, C, and F) and the atom it is bound to. When a molecule consists of more than one atom of the investigated type, we display their averaged charge. The individual values are listed in the Supporting Information.

A set of Li-, H-, C-, and F-containing molecules with varying electronegativity differences is used to test and validate the Berry population analysis (BPA). As references, the Mulliken^5^ (MPA) and the atomic-polar-tensor or dipole population analysis^17^ (DPA) was used, since these methods can easily be adapted to molecules in a magnetic field. Note that the computational costs of BPA and DPA are significantly larger than MPA since they require derivatives with respect to nuclear displacements. For every molecule, we calculate the rotational average of the BPA charges [*q*_*I*_^B^(**R**)], MPA charges [*q*_*I*_^*M*^(**R**)], and DPA charges [*q*_*I*_^D^(**R**)]. The results of each series are in Figure 4 plotted against the corresponding Pauling electronegativity^65^ differences (*Δχ*).

We begin with a brief comparison of the two established population analyses. In all four series, the behavior of the DPA is significantly different from the behavior of the MPA. The DPA covers a wider range of charges and increases monotonically with *Δχ*, while the MPA curves are more bumpy. These results are not surprising given the conceptual differences between the approaches. While we do not consider DPA and MPA methods to yield “exact” charges at our chosen level of theory (the MPA charges are notorious for their basis-set dependence and the DPA charges suffer from erratic dipole moments at the HF level of theory), the DPA charges appear to be more natural in our test cases.

Interestingly, the BPA charges are in good agreement with the DPA charges in all four series, but differ significantly from the MPA charges. The average difference between the BPA and DPA charges is less than 0.05 *e*, while it is about 0.2 *e* between the BPA and the MPA charges. The BPA and DPA charges thus show the same reasonable behavior with increasing electronegativity differences.

**Figure 5:**
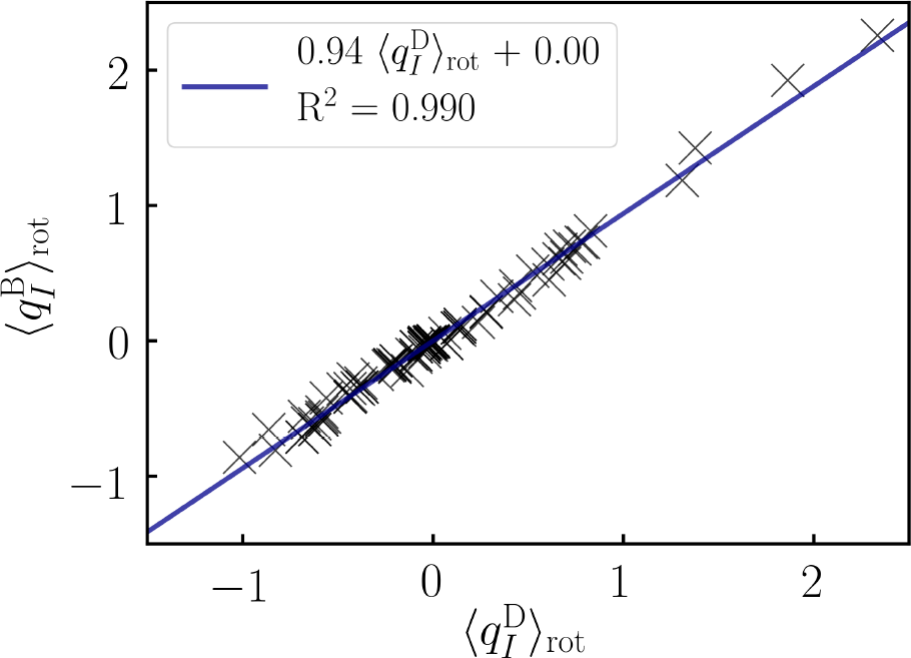
Linear regression between all rotationally averaged Berry (B) and atomic-polar-tensor (D) atomic charges (*q*_*I*_^B/D^, in *e*) calculated in this work. The individual values are listed in the Supporting Information.

The absolute values of the BPA charges are slightly smaller than those of the DPA charges (see Figure 5), where the slope of the linear regression between the two charges is 0.94. One possible reason for this difference is that the DPA charges include charge-transfer and/or polarization contributions, which are only partially included in the BPA charges, if at all.^66^ This explanation is supported by the observation that the Berry charge fluctuations and the total dipole moment tend to be larger when the BPA and the DPA charges differ more strongly.

**Figure 6:**
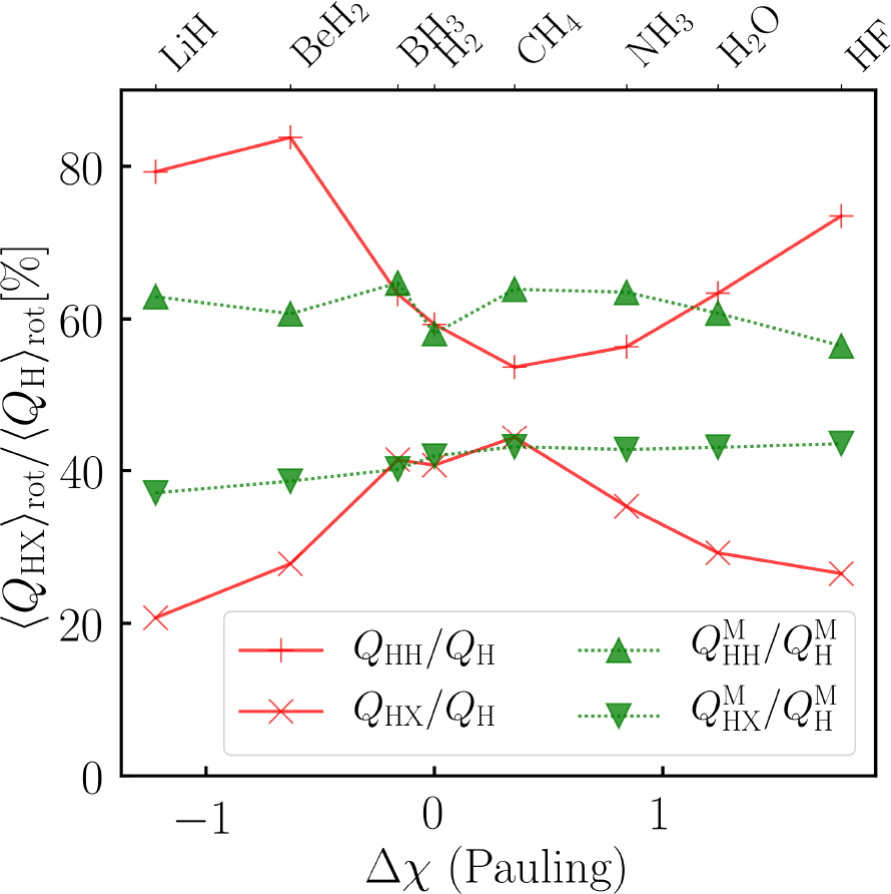
Comparison of rotationally averaged Berry charges (*Q*_HX_) and Mulliken overlap populations (*Q*_HX_^M^, see eq 55) for a series of H-containing molecules XH_*n*_ (X = H, Li–F). For comparison, we divide both by the corresponding charges (*Q*_H_ = *q*_H_^B^ – *Z*_H_; *Q*_H_^M^ = *q*_H_^M^ – *Z*_H_) of hydrogen. The molecules are sorted according to the Pauling electronegativity difference (*Δχ*) between X and H. When a molecule consists of more than one hydrogen atom, we display averaged values.

Finally, we compare the rotationally averaged Berry charges with the Mulliken overlap populations. In Figure 6, we plot these quantities divided by the atomic charges (without *Z*_H_) for a series of H-containing molecules of type XH_*n*_. In this way, we obtain a measure for how much of the atomic charge on hydrogen stems from the atom itself [*Q*_HH_(**R**)/*Q*_H_(**R**)] and from the neighboring atom X [*Q*_HX_(**R**)/*Q*_H_(**R**)] in the BPA and MPA schemes. A similar quantity cannot be obtained from the DPA without further assumptions.

The BPA results agree with chemical intuition. For the ionic molecules LiH and FH, more than 70% of the atomic charge of hydrogen can be assigned to hydrogen itself. This value decreases significantly as the HX bond becomes more covalent, reaching a minimum at 53% for CH_4_. The latter value correlates with the idea that two atoms “share” electrons in a covalent bond. This trend is not observed for the MPA, where the values are almost the same (about 60%) for all molecules. The MPA thus allows bonds to be identified but cannot distinguish between covalent and ionic bonds, unlike the BPA.

## 5 Conclusions and Outlook

In this work, we have shown that the Berry curvature can be rewritten in terms of the magnetic field and charge-like contributions as its role in the screening of the nuclei by electrons implies. The resulting Berry charges and charge fluctuations capture a large part of the exact Berry curvature, making them good starting points for further approximations that aim at reducing the computational cost of *ab initio* molecular dynamics in a strong magnetic field. Additionally, these charges can be used to construct atomic charges and overlap populations. The first results of this Berry population analysis (BPA) were encouraging, since the atomic charges were physically reasonable and close to the results of the widely used and well-established atomic-polar-tensor (DPA) charges. In addition, the overlap populations, which are not accessible in the DPA, give insight into the binding modes (covalent/ionic), while the Berry charge fluctuations indicate cases where a simple interpretation of the electronic structure in terms of atomic charges breaks down. We conclude that the BPA is a promising tool for the investigation of molecules, from which we expect to gain new insight into chemical bonds and electronic structure with and without a magnetic field.

## Appendix: Eigenvalues of the General Λ-Matrix

We begin by introducing a coordinate system depending on the normalized interatomic distance vector **R̅**_*IJ*_ and the magnetic field **B**:

63

Dropping the explicit dependence on **R**, we rewrite the matrices **B** and **Γ**_*IJ*_^+^ in terms of these vectors using the angle θ between **R̅**_*IJ*_ and **B**:

64

65

so that **Ω**_*IJ*_^B2^ takes the following form:

66

From this, we can now obtain the eigenvalues of

67

as:

68

69

## Supporting Information Available

Convergence study of the numerical spherical integration and list of all atomic charges plotted in this work(PDF)

## References

[ref1] Wiberg K. B.; Rablen P. R. Comparison of atomic charges derived via different procedures. J. Comput. Chem. 1993, 14, 1504–1518. 10.1002/jcc.540141213.

[ref2] Meister J.; Schwarz W. H. E. Principal Components of Ionicity. J. Chem. Phys. 1994, 98, 8245–8252. 10.1021/j100084a048.

[ref3] Cramer C. J. Essentials of Computational Chemistry: Theories and Models; Wiley, 2004.

[ref4] Cho M.; Sylvetsky N.; Eshafi S.; Santra G.; Efremenko I.; Martin J. M. The Atomic Partial Charges Arboretum: Trying to See the Forest for the Trees. ChemPhysChem 2020, 21, 688–696. 10.1002/cphc.202000040. 32052532 PMC7317385

[ref5] Mulliken R. S. Electronic Population Analysis on LCAO–MO Molecular Wave Functions. I. J. Chem. Phys. 1955, 23, 1833 10.1063/1.1740588.

[ref6] Mulliken R. S. Electronic Population Analysis on LCAO–MO Molecular Wave Functions. II. Overlap Populations, Bond Orders, and Covalent Bond Energies. J. Chem. Phys. 1955, 23, 1841–1846. 10.1063/1.1740589.

[ref7] Mulliken R. S. Electronic population analysis on LCAO–MO molecular wave functions. III. effects of hybridization on overlap and gross AO populations. J. Chem. Phys. 1955, 23, 2338–2342. 10.1063/1.1741876.

[ref8] Löwdin P.-O. On the non-orthogonality problem connected with the use of atomic wave functions in the theory of molecules and crystals. J. Chem. Phys. 1950, 18, 365–375. 10.1063/1.1747632.

[ref9] Baker J. Classical chemical concepts from ab initio SCF calculations. Theor. Chim. Acta 1985, 68, 221–229. 10.1007/BF00526773.

[ref10] Reed A. E.; Weinstock R. B.; Weinhold F. Natural population analysis. J. Chem. Phys. 1985, 83, 735–746. 10.1063/1.449486.

[ref11] Maslen E. N.; Spackman M. A. Atomic Charges and Electron Density Partitioning. Aust. J. Phys. 1985, 38, 273 10.1071/PH850273.

[ref12] Hirshfeld F. L. Bonded-atom fragments for describing molecular charge densities. Theor. Chim. Acta 1977, 44, 129–138. 10.1007/BF00549096.

[ref13] Marenich A. V.; Jerome S. V.; Cramer C. J.; Truhlar D. G. Charge model 5: An extension of hirshfeld population analysis for the accurate description of molecular interactions in gaseous and condensed phases. J. Chem. Theory Comput. 2012, 8, 527–541. 10.1021/ct200866d. 26596602

[ref14] Manz T. A.; Limas N. G. Introducing DDEC6 atomic population analysis: Part 1. Charge partitioning theory and methodology. RSC Adv. 2016, 6, 47771–47801. 10.1039/C6RA04656H. PMC9096813 35703680

[ref15] Limas N. G.; Manz T. A. Introducing DDEC6 atomic population analysis: Part 2. Computed results for a wide range of periodic and nonperiodic materials. RSC Adv. 2016, 6, 45727–45747. 10.1039/C6RA05507A.

[ref16] Bayly C. I.; Cieplak P.; Cornell W. D.; Kollman P. A. A well-behaved electrostatic potential based method using charge restraints for deriving atomic charges: The RESP model. J. Phys. Chem. 1993, 97, 10269–10280. 10.1021/j100142a004.

[ref17] Cioslowski J. A new population analysis based on atomic polar tensors. J. Am. Chem. Soc. 1989, 111, 8333–8341. 10.1021/ja00204a001.

[ref18] Cioslowski J.; Hamilton T.; Scuseria G.; Hess B. A.; Hu J.; Schaad L. J.; Dupuis M. Application of the GAPT Population Analysis to Some Organic Molecules and Transition Structures. J. Am. Chem. Soc. 1990, 112, 4183–4186. 10.1021/ja00167a012.

[ref19] Haaland A.; Helgaker T.; Ruud K.; Shorokhov D. J. Should gaseous BF3 amd SiF4 be described as iopnic compounds. J. Chem. Educ. 2000, 77, 1076–1080. 10.1021/ed077p1076.

[ref20] Shukla A. Ab initio Hartree-Fock Born effective charges of LiH, LiF, LiCl, NaF, and NaCl. Phys. Rev. B 2000, 61, 13277–13282. 10.1103/PhysRevB.61.13277.

[ref21] Milani A.; Castiglioni C. Atomic charges from atomic polar tensors: A comparison of methods. J. Mol. Struct. THEOCHEM 2010, 955, 158–164. 10.1016/j.theochem.2010.06.011.

[ref22] Peters L. D. M.; Culpitt T.; Monzel L.; Tellgren E. I.; Helgaker T. Ab Initio molecular dynamics with screened Lorentz forces. II. Efficient propagators and rovibrational spectra in strong magnetic fields. J. Chem. Phys. 2021, 155, 024105 10.1063/5.0056235. 34266256

[ref23] Monzel L.; Pausch A.; Peters L. D. M.; Tellgren E. I.; Helgaker T.; Klopper W. Molecular dynamics of linear molecules in strong magnetic fields. J. Chem. Phys. 2022, 157, 054106 10.1063/5.0097800. 35933207

[ref24] Tellgren E. I.; Soncini A.; Helgaker T. Nonperturbative ab initio calculations in strong magnetic fields using London orbitals. J. Chem. Phys. 2008, 129, 154114 10.1063/1.2996525. 19045183

[ref25] Tellgren E. I.; Helgaker T.; Soncini A. Non-perturbative magnetic phenomena in closed-shell paramagnetic molecules. Phys. Chem. Chem. Phys. 2009, 11, 5489–5498. 10.1039/b822262b. 19551219

[ref26] Lange K. K.; Tellgren E. I.; Hoffmann M. R.; Helgaker T. Mechanism for Diatomics in. Science (80-.) 2012, 337, 327–332. 10.1126/science.1219703. 22822146

[ref27] Tellgren E. I.; Reine S. S.; Helgaker T. Analytical GIAO and hybrid-basis integral derivatives: Application to geometry optimization of molecules in strong magnetic fields. Phys. Chem. Chem. Phys. 2012, 14, 9492–9499. 10.1039/c2cp40965h. 22653039

[ref28] Reynolds R. D.; Shiozaki T. Fully relativistic self-consistent field under a magnetic field. Phys. Chem. Chem. Phys. 2015, 17, 14280–14283. 10.1039/C4CP04027A. 25310527

[ref29] Stopkowicz S.; Gauss J.; Lange K. K.; Tellgren E. I.; Helgaker T. Coupled-cluster theory for atoms and molecules in strong magnetic fields. J. Chem. Phys. 2015, 143, 074110 10.1063/1.4928056. 26298118

[ref30] Hampe F.; Stopkowicz S. Equation-of-motion coupled-cluster methods for atoms and molecules in strong magnetic fields. J. Chem. Phys. 2017, 146, 154105 10.1063/1.4979624. 28433009

[ref31] Irons T. J. P.; Zemen J.; Teale A. M. Efficient Calculation of Molecular Integrals over London Atomic Orbitals. J. Chem. Theory Comput. 2017, 13, 3636–3649. 10.1021/acs.jctc.7b00540. 28692291

[ref32] Hampe F.; Stopkowicz S. Transition-Dipole Moments for Electronic Excitations in Strong Magnetic Fields Using Equation-of-Motion and Linear Response Coupled-Cluster Theory. J. Chem. Theory Comput. 2019, 15, 4036–4043. 10.1021/acs.jctc.9b00242. 31141671

[ref33] Sen S.; Lange K. K.; Tellgren E. I. Excited States of Molecules in Strong Uniform and Nonuniform Magnetic Fields. J. Chem. Theory Comput. 2019, 15, 3974–3990. 10.1021/acs.jctc.9b00103. 31117478

[ref34] Sun S.; Williams-Young D. B.; Stetina T. F.; Li X. Generalized Hartree-Fock with Nonperturbative Treatment of Strong Magnetic Fields: Application to Molecular Spin Phase Transitions. J. Chem. Theory Comput. 2019, 15, 348–356. 10.1021/acs.jctc.8b01140. 30485745

[ref35] Austad J.; Borgoo A.; Tellgren E. I.; Helgaker T. Bonding in the helium dimer in strong magnetic fields: The role of spin and angular momentum. Phys. Chem. Chem. Phys. 2020, 22, 23502–23521. 10.1039/D0CP03259J. 33078796

[ref36] Hampe F.; Gross N.; Stopkowicz S. Full triples contribution in coupled-cluster and equation-of-motion coupled-cluster methods for atoms and molecules in strong magnetic fields. Phys. Chem. Chem. Phys. 2020, 22, 23522–23529. 10.1039/D0CP04169F. 33078770

[ref37] Pausch A.; Klopper W. Efficient evaluation of three-centre two-electron integrals over London orbitals. Mol. Phys. 2020, 118, e1736675 10.1080/00268976.2020.1736675.

[ref38] Williams-Young D. B.; Petrone A.; Sun S.; Stetina T. F.; Lestrange P.; Hoyer C. E.; Nascimento D. R.; Koulias L.; Wildman A.; Kasper J.; Goings J. J.; Ding F.; DePrince A. E.; Valeev E. F.; Li X. The Chronus Quantum software package. Wiley Interdiscip. Rev. Comput. Mol. Sci. 2020, 10, e1436 10.1002/wcms.1436.

[ref39] Irons T. J. P.; David G.; Teale A. M. Optimizing Molecular Geometries in Strong Magnetic Fields. J. Chem. Theory Comput. 2021, 17, 2166–2185. 10.1021/acs.jctc.0c01297. 33724812 PMC8047810

[ref40] Blaschke S.; Stopkowicz S. Cholesky decomposition of complex two-electron integrals over GIAOs: Efficient MP2 computations for large molecules in strong magnetic fields. J. Chem. Phys. 2022, 156, 044115 10.1063/5.0076588. 35105060

[ref41] London F. Quantum theory of interatomic currents in aromatic compounds. J. Phys. Radium 1937, 8, 397–409. 10.1051/jphysrad:01937008010039700.

[ref42] Hameka H. F. On the nuclear magnetic shielding in the hydrogen molecule. Mol. Phys. 1958, 1, 203–215. 10.1080/00268975800100261.

[ref43] Ditchfield R. Theoretical studies of magnetic shielding in H2O and (H2O)2. J. Chem. Phys. 1976, 65, 3123–3133. 10.1063/1.433526.

[ref44] Helgaker T.; Jørgensen P. An electronic Hamiltonian for origin independent calculations of magnetic properties. J. Chem. Phys. 1991, 95, 2595–2801. 10.1063/1.460912.

[ref45] Culpitt T.; Peters L. D. M.; Tellgren E. I.; Helgaker T. Ab initio molecular dynamics with screened Lorentz forces. I. Calculation and atomic charge interpretation of Berry curvature. J. Chem. Phys. 2021, 155, 024104 10.1063/5.0055388. 34266267

[ref46] Peters L. D. M.; Culpitt T.; Tellgren E. I.; Helgaker T. Magnetic-translational sum rule and approximate models of the molecular Berry curvature. J. Chem. Phys. 2022, 157, 134108 10.1063/5.0112943. 36208997

[ref47] Schmelcher P.; Cederbaum L. S.; Meyer H. D. Electronic and nuclear motion and their couplings in the presence of a magnetic field. Phys. Rev. A 1988, 38, 6066–6079. 10.1103/PhysRevA.38.6066. 9900362

[ref48] Schmelcher P.; Cederbaum L. S. Approximate constant of motion for molecular ions in a magnetic field. Phys. Rev. A 1989, 40, 3515–3523. 10.1103/PhysRevA.40.3515. 9902565

[ref49] Yin L.; Mead C. A. Magnetic screening of nuclei by electrons as manifestation of geometric vector potential. Theor. Chim. Acta 1992, 82, 397–406. 10.1007/BF01113940.

[ref50] Peternelj J.; Kranjc T. Nonadiabatic screening of proton charges in the case of tunneling methyl groups in external magnetic field. Z. Phys. B 1993, 92, 61–66. 10.1007/BF01309168.

[ref51] Yin L.; Mead C. A. Magnetic screening of nuclei by electrons as manifestation of geometric vector potential. J. Chem. Phys. 1994, 100, 8125 10.1063/1.466806.

[ref52] Schmelcher P.; Cederbaum L. S. Molecules in strong magnetic fields: Some perspectives and general aspects. Int. J. Quantum Chem. 1997, 64, 501–511. 10.1002/(SICI)1097-461X(1997)64:5<501::AID-QUA3>3.0.CO;2-#.

[ref53] Ceresoli D.; Marchetti R.; Tosatti E. Electron-corrected Lorentz forces in solids and molecules in a magnetic field. Phys. Rev. B 2007, 75, 161101 10.1103/PhysRevB.75.161101.

[ref54] Berry M. V. Quantal phase factors accompanying adiabatic changes. Proc. R. Soc. London A 1984, 392, 45–57. 10.1098/rspa.1984.0023.

[ref55] Mead C. A. The geometric phase in molecular systems. Rev. Mod. Phys. 1992, 64, 51–85. 10.1103/RevModPhys.64.51.

[ref56] Anandan J.; Christian J.; Wanelik K. Resource Letter GPP-1: Geometric Phases in Physics. Am. J. Phys. 1997, 65, 180–185. 10.1119/1.18570.

[ref57] Resta R. Manifestations of Berry’s phase in molecules and condensed matter. J. Phys.: Condens. Matter 2000, 12, R107–R143. 10.1088/0953-8984/12/9/201.

[ref58] Culpitt T.; Peters L. D. M.; Tellgren E. I.; Helgaker T. Analytic calculation of the Berry curvature and diagonal Born – Oppenheimer correction for molecular systems in uniform magnetic fields. J. Chem. Phys. 2022, 156, 044121 10.1063/5.0079304. 35105065

[ref59] Zabalo A.; Dreyer C. E.; Stengel M. Rotational g factors and Lorentz forces of molecules and solids from density functional perturbation theory. Phys. Rev. B 2022, 105, 094305 10.1103/PhysRevB.105.094305.

[ref60] Smith S. A.; Palke W. E.; Gerig J. T. The Hamiltonians of NMR. Concepts Magn. Reson. 1992, 4, 107 10.1002/cmr.1820040202.

[ref61] Pausch A. I. Development and Application of Efficient Computational Methods for Molecular Spectroscopy in Finite Magnetic Fields. Ph.D. thesis, Karlsruher Institut für Technologie (KIT), 2022.

[ref62] Tellgren E.; Helgaker T.; Soncini A.; Lange K. K.; Teale A. M.; Ekström U.; Stopkowicz S.; Austad J. H.; Se S. LONDON, a quantum-chemistry program for plane-wave/GTO hybrid basis sets and finite magnetic field calculations.

[ref63] Dunning T. H. Gaussian basis sets for use in correlated molecular calculations. I. The atoms boron through neon and hydrogen. J. Chem. Phys. 1989, 90, 1007–1023. 10.1063/1.456153.

[ref64] Schlömer N. quadpy 0.16.14, numerical integration, quadrature for various domains.

[ref65] Pauling L. The nature of the chemical bond IV. J. Am. Chem. Soc. 1932, 54, 3570–3582. 10.1021/ja01348a011.

[ref66] Richter W. E.; Duarte L. J.; Bruns R. E. Are “GAPT Charges” Really Just Charges?. J. Chem. Inf. Model. 2021, 61, 3881–3890. 10.1021/acs.jcim.1c00165. 34324335 PMC8391781

