# Supplementary material for: Berry Population Analysis: Atomic Charges from the Berry Curvature in a Magnetic Field
Source: J Chem Theory Comput. 2023 Jan 27;19(4):1231–42. doi: 10.1021/acs.jctc.2c01138 (PMC9979605; doi:10.1021/acs.jctc.2c01138)
Supplement: Supplementary file 1 — ct2c01138_si_001.pdf [file ct2c01138_si_001.pdf]

Berry Population Analysis:
Atomic Charges from the Berry Curvature in a Magnetic Field
# – Supporting Information

Laurens D. M. Peters,[1, a)] Tanner P. Culpitt,[1] Erik I. Tellgren,[1] and Trygve Helgaker[1]

Hylleraas Centre for Quantum Molecular Sciences, Department of Chemistry, University of Oslo, P.O. Box 1033 Blindern, N-0315 Oslo, Norway

(Dated: 3 January 2023)

[a)] Electronic mail: laurens.peters@kjemi.uio.no

CONTENTS

I. Convergence of Numerical Spherical Integration 3

II. Rotationally Averaged Atomic Charges 4

References 8

I. CONVERGENCE OF NUMERICAL SPHERICAL INTEGRATION

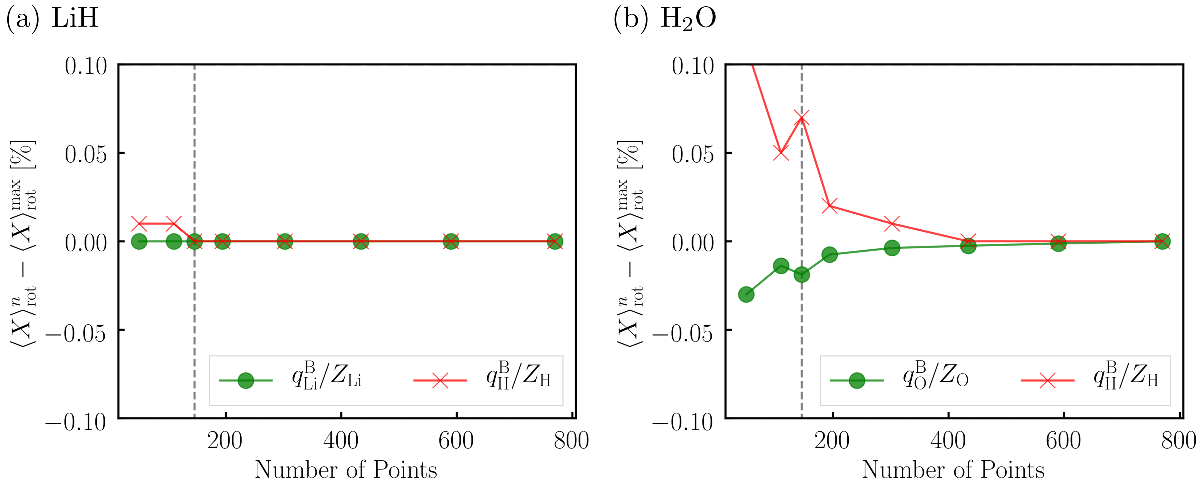

FIG. 1: Convergence of rotationally averaged charges of LiH (a) and H_2O (b) with the number of grid points n for the numerical spherical integration. All calculations were performed at the HF/cc-pVDZ/$\mathbf{B} = 0.001\ B_0$ level of theory. Grid points and weights were taken from the QUADPY[1] program package. Our desired accuracy and choice for this work are indicated by horizontal and vertical dashed lines, respectively. The final point was set to zero.

II. ROTATIONALLY AVERAGED ATOMIC CHARGES

TABLE I: Rotationally averaged atomic charges (q_I, in e) of different molecules obtained from Berry (B), atomic-polar-tensor (D), and Mulliken (M) population analyses. All values were obtained at the HF/cc-pVDZ/$\mathbf{B} = 0.001\ B_0$ level of theory using 146 grid points/weights taken from the QUADPY[1] program package.

Molecule	I	$\langle q_I^{\mathrm{B}} \rangle$	$\langle q_I^{\mathrm{D}} \rangle$	$\langle q_I^{\mathrm{M}} \rangle$
LiF	F	-0.8109	-0.8270	-0.6227
	Li	0.8110	0.8270	0.6227
BeF_2	Be	1.4251	1.3766	0.8211
	F	-0.7124	-0.6883	-0.4105
	F	-0.7124	-0.6883	-0.4105
BF_3	B	1.9261	1.8620	0.6142
	F	-0.6121	-0.6207	-0.2047
	F	-0.6568	-0.6207	-0.2047
	F	-0.6568	-0.6207	-0.2047
CF_4	F	-0.5641	-0.5837	-0.2059
	C	2.2568	2.3346	0.8235
	F	-0.5257	-0.5837	-0.2059
	F	-0.5832	-0.5837	-0.2059
	F	-0.5832	-0.5837	-0.2059
NH_3	N	1.1867	1.3094	0.5756
	F	-0.3542	-0.4365	-0.1919
	F	-0.4160	-0.4365	-0.1919
	F	-0.4160	-0.4365	-0.1919
OF_2	O	0.3526	0.4545	0.2158
	F	-0.1761	-0.2273	-0.1079
	F	-0.1761	-0.2273	-0.1079
F_2	F	0.0001	-0.0000	0.0000
	F	0.0001	-0.0000	0.0000
$LiCH_3$	C	-0.3462	-0.3998	-0.3230
	Li	0.6761	0.6716	0.3052
	H	-0.1047	-0.0906	0.0059
	H	-0.1047	-0.0906	0.0059
	H	-0.1204	-0.0906	0.0059

TABLE II: Rotationally averaged atomic charges (q_I, in e) of different molecules obtained from Berry (B), atomic-polar-tensor (D), and Mulliken (M) population analyses. All values were obtained at the HF/cc-pVDZ/$\mathbf{B} = 0.001\ B_0$ level of theory using 146 grid points/weights taken from the QUADPY[1] program package.

Molecule	I	$\langle q_I^{\mathrm{B}} \rangle$	$\langle q_I^{\mathrm{D}} \rangle$	$\langle q_I^{\mathrm{M}} \rangle$
$BeHCH_3$	C	-0.3117	-0.3780	-0.1443
	Be	0.7245	0.7902	0.1882
	H	-0.0306	-0.0067	0.0286
	H	-0.0101	-0.0067	0.0286
	H	-0.0136	-0.0067	0.0286
	H	-0.3585	-0.3920	-0.1298
BH_2CH_3	C	-0.1755	-0.2108	-0.1571
	B	0.5735	0.6911	0.1839
	H	-0.0091	0.0027	0.0414
	H	-0.0115	-0.0226	0.0325
	H	-0.0091	0.0027	0.0414
	H	-0.1851	-0.2362	-0.0707
	H	-0.1832	-0.2269	-0.0713
CH_3CH_3	C	0.1224	0.1417	-0.0886
	C	0.1224	0.1417	-0.0886
	H	-0.0487	-0.0472	0.0295
	H	-0.0251	-0.0472	0.0295
	H	-0.0487	-0.0472	0.0295
	H	-0.0251	-0.0472	0.0295
	H	-0.0487	-0.0472	0.0295
	H	-0.0487	-0.0472	0.0295
CH_3NH_2	C	0.3043	0.4270	0.0471
	N	-0.3550	-0.4728	-0.2922
	H	-0.0207	-0.0487	0.0325
	H	-0.0207	-0.0487	0.0325
	H	-0.0970	-0.1114	0.0071
	H	0.0946	0.1273	0.0865
	H	0.0946	0.1273	0.0865
CH_3OH	C	0.4516	0.6094	0.1467
	O	-0.5574	-0.6767	-0.3594
	H	-0.0446	-0.0876	0.0118
	H	-0.0202	-0.0366	0.0386
	H	-0.0446	-0.0876	0.0118
	H	0.2155	0.2791	0.1504
CH_3F	C	0.6176	0.7030	0.2478
	F	-0.5629	-0.5692	-0.3358
	H	-0.0120	-0.0446	0.0293
	H	-0.0304	-0.0446	0.0293
	H	-0.0120	-0.0446	0.0293

TABLE III: Rotationally averaged atomic charges (q_I, in e) of different molecules obtained from Berry (B), atomic-polar-tensor (D), and Mulliken (M) population analyses. All values were obtained at the HF/cc-pVDZ/$\mathbf{B} = 0.001\ B_0$ level of theory using 146 grid points/weights taken from the QUADPY[1] program package.

Molecule	I	$\langle q_I^{\mathrm{B}} \rangle$	$\langle q_I^{\mathrm{D}} \rangle$	$\langle q_I^{\mathrm{M}} \rangle$
LiH	Li	0.6063	0.6604	0.1254
	H	-0.6063	-0.6604	-0.1254
BeH2	Be	0.6795	0.7446	0.2260
	H	-0.3399	-0.3723	-0.1130
	H	-0.3399	-0.3723	-0.1130
BH_3	B	0.5673	0.6041	0.1575
	H	-0.1816	-0.2014	-0.0525
	H	-0.1928	-0.2014	-0.0525
	H	-0.1928	-0.2014	-0.0525
CH_4	H	-0.0138	-0.0156	0.0369
	C	0.0551	0.0624	-0.1477
	H	-0.0271	-0.0156	0.0369
	H	-0.0071	-0.0156	0.0369
	H	-0.0071	-0.0156	0.0369
NH_3	N	-0.2791	-0.4207	-0.2561
	H	0.0783	0.1402	0.0854
	H	0.1005	0.1402	0.0854
	H	0.1005	0.1402	0.0854
H_2O	O	-0.4219	-0.5605	-0.2897
	H	0.2110	0.2802	0.1449
	H	0.2110	0.2802	0.1449
HF	F	-0.4079	-0.4332	-0.2230
	H	0.4081	0.4332	0.2230

TABLE IV: Rotationally averaged atomic charges (q_I, in e) of different molecules obtained from Berry (B), atomic-polar-tensor (D), and Mulliken (M) population analyses. All values were obtained at the HF/cc-pVDZ/$\mathbf{B} = 0.001\ B_0$ level of theory using 146 grid points/weights taken from the QUADPY[1] program package.

Molecule	I	$\langle q_I^{\mathrm{B}} \rangle$	$\langle q_I^{\mathrm{D}} \rangle$	$\langle q_I^{\mathrm{M}} \rangle$
Li_2	Li	0.0013	0.0000	0.0000
	Li	0.0013	0.0000	0.0000
LiBeH	Be	-0.0809	-0.0424	0.1422
	Li	0.3303	0.3358	0.0138
	H	-0.2495	-0.2933	-0.1560
$LiBH_2$	B	-0.2196	-0.1341	0.0635
	Li	0.5141	0.5244	0.1174
	H	-0.1474	-0.1951	-0.0904
	H	-0.1474	-0.1951	-0.0904
$LiNH_2$	N	-0.6570	-0.8624	-0.5295
	Li	0.6992	0.7170	0.4103
	H	-0.0210	0.0727	0.0596
	H	-0.0210	0.0727	0.0596
LiOH	O	-0.8622	-1.0174	-0.6403
	Li	0.7278	0.7786	0.5158
	H	0.1345	0.2388	0.1246

REFERENCES

[1] "quadpy 0.16.14, Numerical integration, quadrature for various domains. By N. Schlömer.".
